# Supplementary material for: Enhancement of thermoelectric properties of La-doped SrTiO3 bulk by introducing nanoscale porosity
Source: R Soc Open Sci. 2019 Oct 23;6(10):190870. doi: 10.1098/rsos.190870 (PMC6837226; doi:10.1098/rsos.190870)
Supplement: XRD, BET Analysis, Electrical and phonon thermal conductivity, Pore size, Lattice parameters and Relative density [file rsos190870supp1.docx]

**Enhancement of Thermoelectric Properties of La-doped SrTiO_3_ Bulk by Introducing Nanoscale Porosity**

Al Jumlat Ahmed,^a^ Sheik Md. Kazi Nazrul Islam,^a^ Ridwone Hossain, ^a^ Jeonghun Kim,^b,c^ Minjun Kim,^c^ Motasim Billah,^c^ Md. Shahriar A. Hossain,*^c,d^ Yusuke Yamauchi^b,c,e,f,g^ and Xiaolin Wang*^a^

* Corresponding authors

^a^ Institute for Superconducting and Electronic Materials (ISEM), Australian Institute of Innovative Materials (AIIM), University of Wollongong, North Wollongong, NSW 2500, Australia, E-mail: xiaolin@uow.edu.au

^b^ Key Laboratory of Eco-chemical Engineering, College of Chemistry and Molecular Engineering, Qingdao University of Science and Technology (QUST), Qingdao 266042, China

^c^ Australian Institute for Bioengineering and Nanotechnology (AIBN), The University of Queensland, Brisbane, QLD 4072, Australia, E-mail: md.hossain@uq.edu.au

^d^ School of Mechanical and Mining Engineering, Faculty of Engineering, Architecture and Information Technology (EAIT), University of Queensland, St Lucia QLD 4072, Australia

^e^ International Center for Materials Nanoarchitectonics (MANA), National Institute for Materials Science (NIMS), 1-1 Namiki, Tsukuba, Ibaraki 305-0044, Japan

^f^ School of Chemical Engineering, Architecture and Information Technology (EAIT), University of Queensland, St Lucia QLD 4072, Australia

^g^ Department of Plant & Environmental New Resources, Kyung Hee University, 1732 Deogyeong-daero, Giheung-gu, Yongin-si, Gyeonggi-do 446-701, South Korea


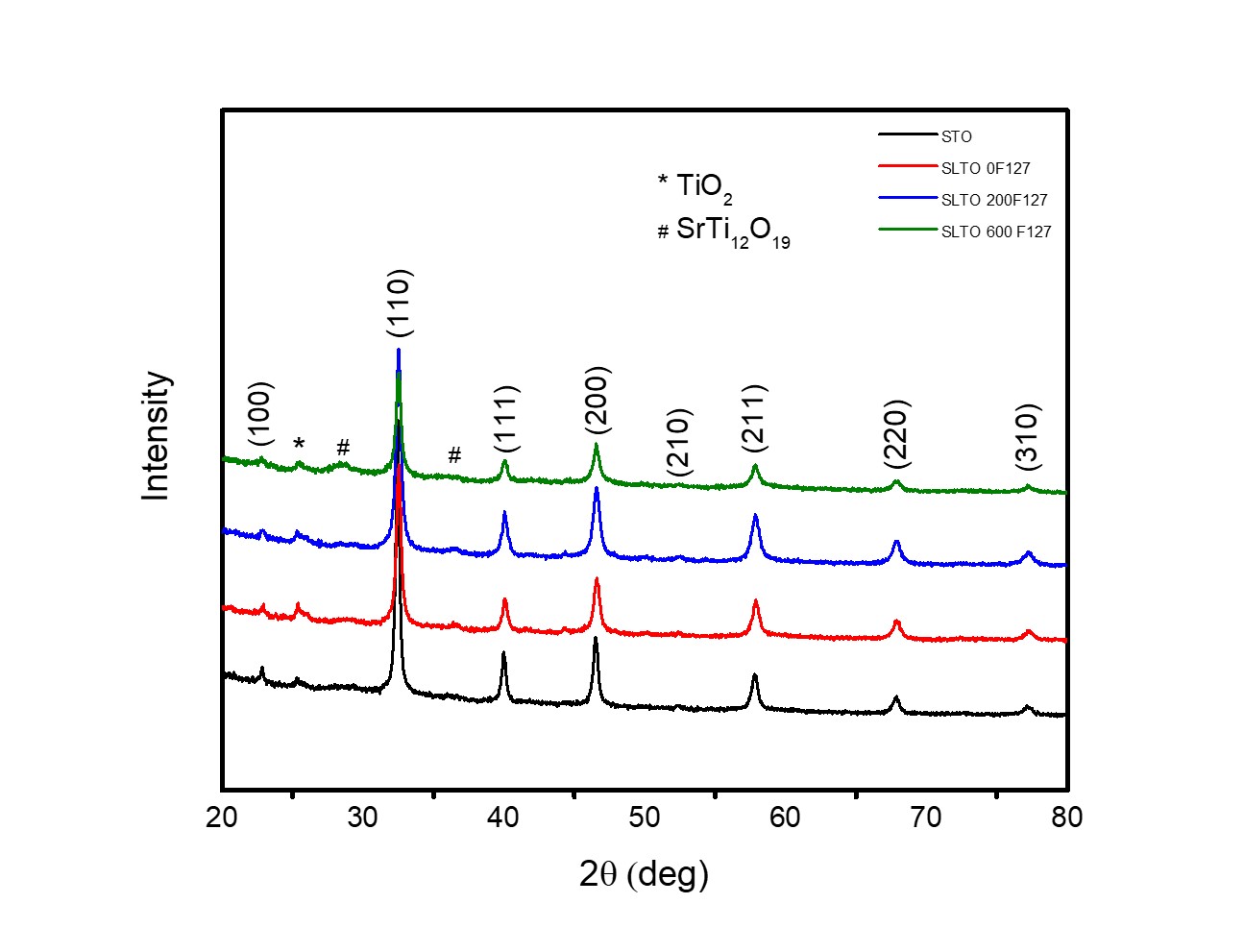


Fig. S1 XRD patterns of La-doped SrTiO_3_ calcinated powders with different amounts of F127 surfactant with reference to undoped SrTiO_3_. There are some impurity phases such as TiO_2_ and SrTi_12_O_19_ ^29^ with peaks in the 2*θ* range of 25^o^ – 35^o^ in the XRD patterns.


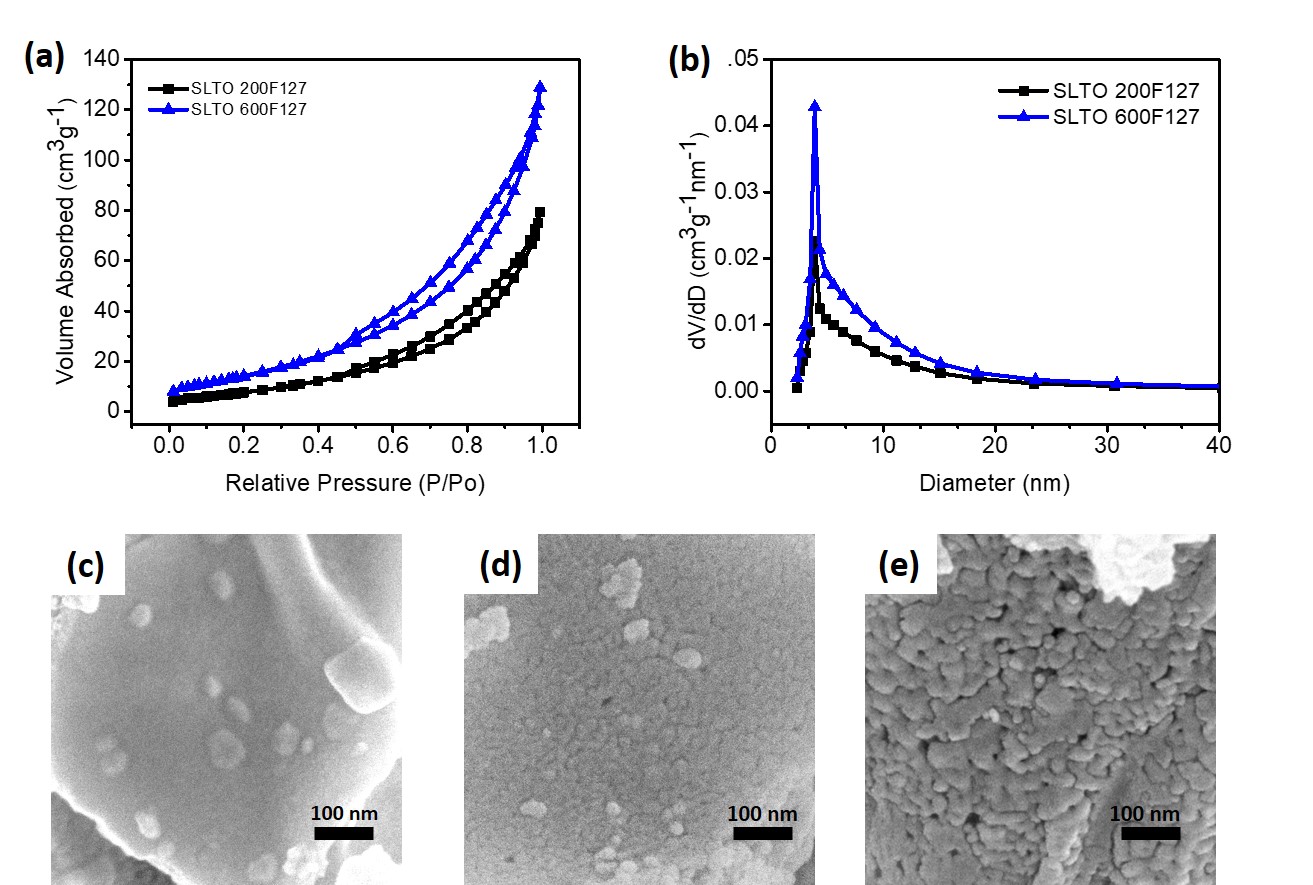


Fig. S2 (a) Nitrogen absorption/desorption isotherms, and (b) pore size distributions of La-doped SrTiO_3_ calcinated powders with different amounts of F127 surfactant. SEM images of La-doped SrTiO_3_ calcinated powders with different amounts of F127 surfactant: (c) SLTO 0F127, (d) SLTO 200F127, (e) SLTO 600F127.


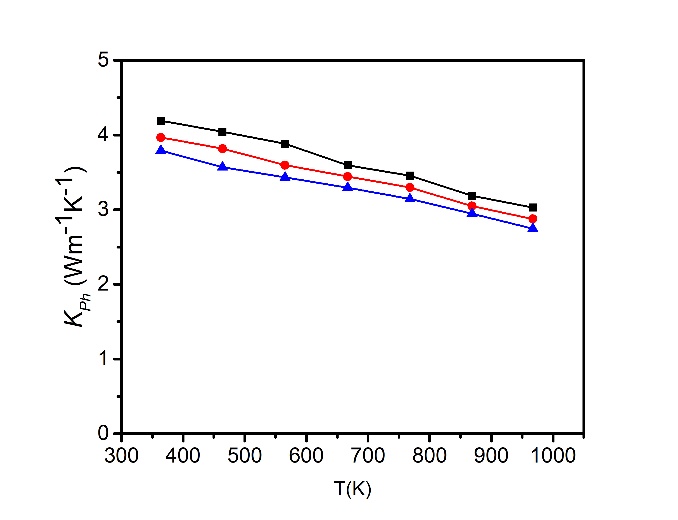

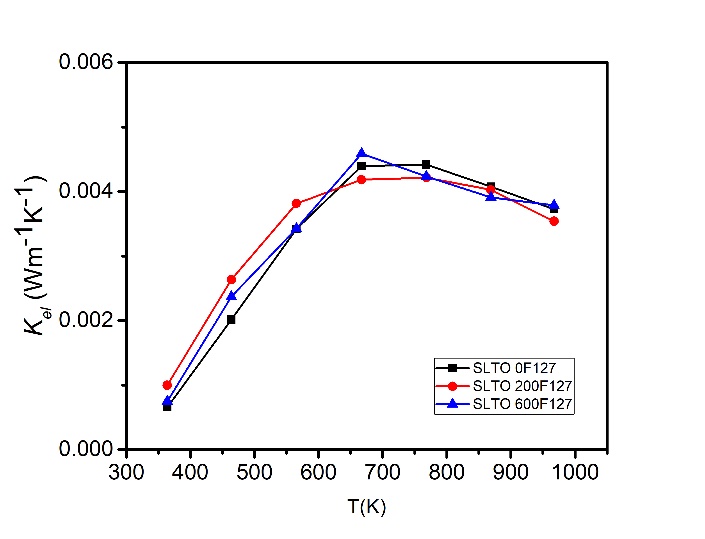


**(a)**

**(b)**

Fig S3 (a) The electronic thermal conductivity and (b) The phonon thermal conductivity of samples with different amount of surfactant F127.

Table S1 Specific surface area, pore volume, and average pore width of samples with different amounts of F127 surfactant

| Sample | Specific Surface Area (m^2^/g) | Pore Volume  (cm^3^/g) | Average Pore Size (nm) |
| --- | --- | --- | --- |
| SLTO 200F127 | 31.35 | 0.143 | 8.75 |
| SLTO 600F127 | 56.81 | 0.231 | 8.41 |

Table S2 Lattice parameter of samples with different amount of F127 surfactant

| Sample | Lattice Parameter (nm) |
| --- | --- |
| STO | 0.3901 |
| SLTO 0F127 | 0.3891 |
| SLTO 200F127 | 0.3894 |
| SLTO 600F127 | 0.3889 |

Table S3 Density and relative density of samples with different amounts of F127 surfactant

| Sample name | Amount of F127 (mg) | Density (g/cm^3^) | Relative Density (%) |
| --- | --- | --- | --- |
| SLTO 0F127 | 0 | 4.81 | 93.95 |
| SLTO 200F127 | 200 | 4.66 | 91.02 |
| SLTO 600F127 | 600 | 4.51 | 88.09 |
